# Effects of *Saccharomyces cerevisiae* on the regulation of skatole, microorganisms, and tryptophan metabolites *in vitro* fermentation of pig

**DOI:** 10.1128/spectrum.03292-25

**Published:** 2026-02-27

**Authors:** Xiaoli Gao, Shengwei Zhang, Shanshan Ge, Qiaoli Yang, Shuangbao Gun

**Affiliations:** 1 College of Animal Science and Technology, Gansu Agricultural University 74661, Lanzhou, China; 2 Farmer Education and Training Work Station of Gansu Province, Lanzhou, China; 3 Gansu Innovations Center for Swine Production Engineering and Technology, Lanzhou, China; Washington State University, Pullman, Washington, USA

**Keywords:** *Saccharomyces cerevisiae*, skatole, tryptophan metabolism, gut microbiota, pig

## Abstract

Skatole (3-methylindole), one of the most malodorous compounds from swine manure, poses serious risks to animal, human, and environmental health. Therefore, it has become a serious concern in livestock farming. *Saccharomyces cerevisiae* is widely used in livestock feed to improve growth and intestinal health. However, its role in reducing skatole production and the underlying mechanisms remains unclear. To explore this idea, an *in vitro* fermentation study was conducted using pig rectal contents. We evaluated skatole levels by high-performance liquid chromatography and profiled microbiota and metabolites using 16S rRNA sequencing and ultra-high-performance liquid chromatography–tandem mass spectrometry. *S. cerevisiae* supplementation significantly reduced pH and skatole concentration in the fermentation broth. There was a significant increase in the abundance of *Lysinibacillus*, *Sojibacillus, Bacillus*, *Sphingobacterium*, *Moraxella*, and *Proteiniclasticum*, along with a significant decrease in *Peptostreptococcus*, *Prevotellaceae_NK3B31_grou*p, *Prevotellaceae_UCG_001*, *Enterococcus*, *Desulfovibrio*, *Veillonella*, and *Streptococcus*. The abundance of *Sphingobacterium*, *Moraxella*, and *Proteiniclasticum* was negatively correlated with pH and skatole, whereas *Desulfovibrio* and *Streptococcus* showed a positive correlation. Metabolomic analysis revealed that methyl (indol-3-yl)acetate, indoxyl sulfate, and 3-methylindole were significantly downregulated. Meanwhile, 5-hydroxyindole-2-carboxylic acid, Dl-indole-3-lactic acid, 5-hydroxyindole, 4-aminoindole, 5-hydroxytryptophol, and indole-3-acetic acid were significantly upregulated. The levels of 3-methylindole were positively correlated with indoxyl sulfate and negatively correlated with Dl-indole-3-lactic acid and 5-hydroxytryptophol. These results suggest that *S. cerevisiae* reduces skatole amounts via modulation of specific microbial populations and tryptophan metabolism. This study provides a theoretical basis for developing microbial strategies to reduce odor in pig farming.

**IMPORTANCE:**

Skatole is one of the most odorous compounds in the gastrointestinal tract and feces of animals, which poses a serious threat to animal, human, and environmental health. Therefore, taking measures to reduce skatole emissions is essential for sustainable livestock development. *Saccharomyces cerevisiae* as a beneficial feed additive can not only improve feed digestibility and growth performance, reduce pathogenic bacteria, and improve animal health, but also reduce odor emissions from livestock and reduce the impact of livestock production on the environment. This study explored the role of *S. cerevisiae* in reducing skatole production and the underlying mechanisms. The result showed that *S. cerevisiae* reduced skatole content by regulating specific microbial populations and tryptophan metabolism. These findings provide a theoretical reference for developing more effective microbial agents to reduce odor emissions in pig farming.

## INTRODUCTION

Large amounts of malodorous gas are produced in intensive livestock and poultry industries. This gas accumulates rapidly and cannot be processed efficiently, which seriously reduces livestock production performance (1, 2) and pollutes the environment (3). Furthermore, high levels of odorous emissions expose farm workers and nearby residents to respiratory distress and illnesses, such as headache, nausea, convulsions, and other adverse health effects (4, 5). Therefore, malodorous pollution has gained significant attention, and urgent solutions for gas emission control are needed.

Skatole (3-methylindole, C₉H₉N), one of the most malodorous compounds in the gastrointestinal tract and feces of animals, is produced by microbial fermentation of L-tryptophan in the hindgut (6). Skatole has a strong fecal odor and high diffusion ability. Its olfactory threshold is extremely low, below 0.03 μg/m³ in air and approximately 1 μg/L in liquid (7). Previous studies showed that skatole is a key odorant emitted from swine manure during pig farming (8). Its concentration in manure can reach up to 72.2 mg/kg (9). The compound is difficult to biodegrade, persisting in the environment and causing pollution (10). In addition, skatole has moderate toxicity. It may impair intestinal epithelial cells and promote intestinal disease (11). It can induce lung cell necrosis, edema, and emphysema (12). More seriously, skatole can cause DNA damage in bronchial epithelial cells and lung cells, increasing the risk of lung cancer (13). Skatole also accumulates in adipose tissue due to its lipophilic nature, reducing pork quality (14). Due to these harmful effects, skatole has become a significant challenge in livestock systems (15). Thus, measures to reduce skatole emissions are essential for sustainable livestock development.

Currently, traditional methods for reducing skatole, such as chemical additives and fecal treatment, have drawbacks such as high cost, unstable effectiveness, and poor environmental compatibility (16). Therefore, the use of microbial agents is an efficient and environmentally friendly approach for odor emission reduction. Studies have shown that *Bacillus subtilis* may actively prevent or reduce skatole production by regulating anaerobic fermentation of intestinal microorganisms, which benefits both the environment and animal health (17).

*Saccharomyces cerevisiae* has strong fermentation capacity, high nutritional value, and can reduce toxicity. It is widely used in livestock feed to enhance growth and intestinal health (18). Dietary supplementation with 5 g/kg yeast culture has been reported to improve average daily weight gain, average daily feed intake, and apparent digestibility, dry matter, and crude protein. It also improves villus height and the villus height to crypt depth ratio in the ileum of weaning pigs (19). In weaned piglets, *S. cerevisiae* supplementation increases body weight, daily gain, nutrient digestibility, and Lactobacillus counts (18). *S. cerevisiae* dietary inclusion also improves Lactobacillus levels and reduces *Escherichia coli* counts, thereby lowering NH₃ and H₂S emissions in broilers (20). An *in vitro* rumen fermentation experiment using Hu sheep showed that *S. cerevisiae* reduced pH after 48 h and significantly increased microbial protein, NH₃–N concentrations, and total gas production. It also reduced methane emissions (21). Furthermore, *S. cerevisiae* mixed four exhibited the most significant effect on reducing the off-odor of pig large intestines, such as indole, 2-pentylthiophene, (E)-2-octenal, and 2-methoxy-phenol, which were reduced by 28.1%, 23.90%, 21%, and 22.89%, respectively, after fermentation (22). However, the effect of *S. cerevisiae* on skatole emissions and its underlying mechanisms remains unclear. This study evaluated the effect of *S. cerevisiae* supplementation on skatole using an *in vitro* fermentation experiment and explored potential regulatory mechanisms using microbiomic and metabolomic analyses.

## RESULTS

### Growth curve of *S. cerevisiae* and effect of *S. cerevisiae* on pH value

**Fig 1:**
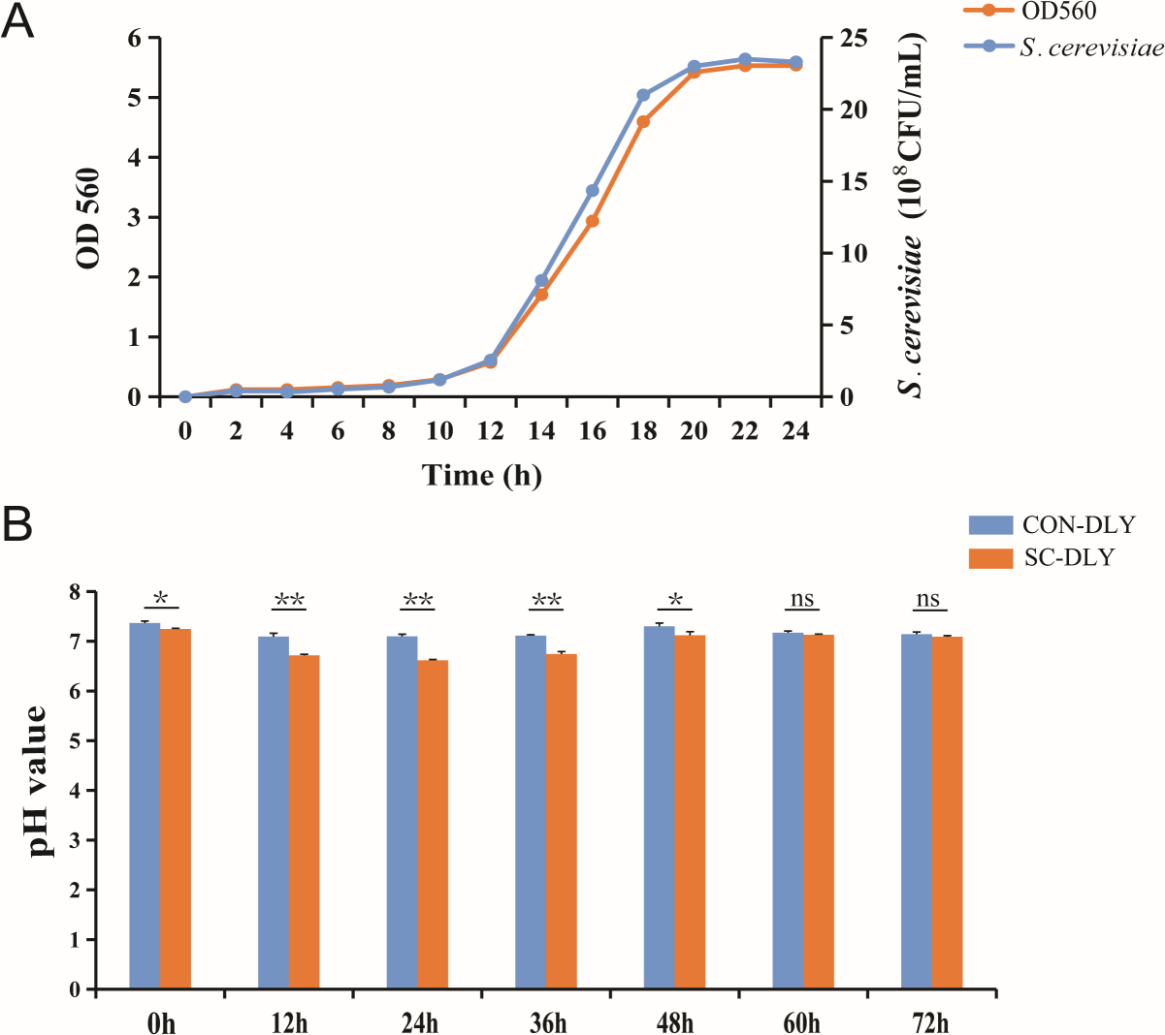
Growth curve of *S. cerevisiae* (**A**) and effect of *S. cerevisiae* on pH value (**B**). CON-DLY: control group, SC-DLY: *S. cerevisiae* supplementation group, the same below. * Indicates a statistically significant difference between the SC-DLY and CON-DLY (*P* < 0.05), ** indicates a statistically highly significant difference between the SC-DLY and CON-DLY (*P* < 0.01), values are mean ± SEM (*n* = 3).

As shown in Fig. 1A, *S*. *cerevisiae* grew slowly from 0 to 10 h and entered the logarithmic growth phase at 10 h. After 22 h, the growth became stable, marking the beginning of a steady phase. During *in vitro* fermentation, the pH value significantly decreased at 0, 12, 24, and 36 h (*P* < 0.01) and 48 h (*P* < 0.05) after *S. cerevisiae* supplementation. The lowest pH value was recorded at 24 h (pH = 6.61), with no significant changes observed at other time points (Fig. 1B). These results indicated that 24 h was the optimal fermentation time. The subsequent experiments were performed based on this finding.

### *S. cerevisiae* reduced the skatole concentration and changed the structure and composition of microbial community during *in vitro* fermentation

**Fig 2:**
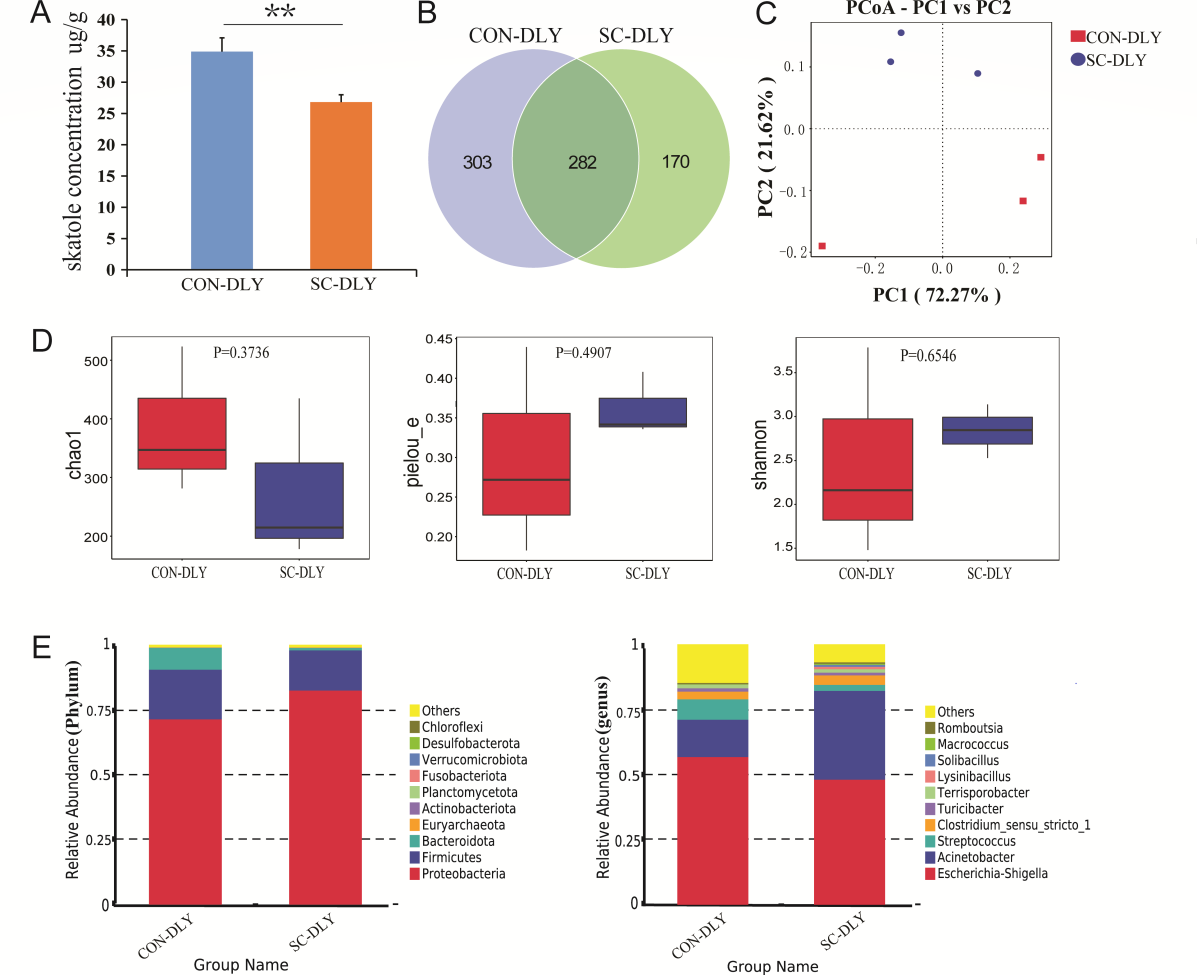
The skatole concentration and microbial community structure and composition during *in vitro* fermentation after *S. cerevisiae* supplementation. (**A**) The skatole concentration between the CON-DLY and SC-DLY groups. ** Indicates a statistically highly significant difference between the SC-DLY and CON-DLY (*P* < 0.01), values are mean ± SEM (*n* = 3). (**B**) Venn diagram of shared and specific OTUs between the CON-DLY and SC-DLY groups. (**C**) Beta diversity on the genera analyzed by Bray–Curtis principal coordinate analysis (PCoA). (**D**) Chao1, Pielou-e, and Shannon indices of alpha diversity between the CON-DLY and SC-DLY groups. (**E**) The top 10 microbial community proportions at the phylum and genus levels between the CON-DLY and SC-DLY groups.

As shown in Fig. 2A, the concentration of skatole in the fermentation solution at 24 h was significantly lower in the SC-DLY group compared to the CON-DLY group (*P* < 0.01), indicating that the addition of *S. cerevisiae* reduced skatole production. 16S rRNA sequencing was employed to reveal microbial community structure and composition. As depicted in the Venn diagram, 755 operational taxonomic units (OTUs) were identified in both groups, while 303 and 170 OTUs were unique to the CON-DLY and SC-DLY groups, respectively (Fig. 2B).

The principal coordinate analysis (PCoA) score plots showed a significant difference between the CON-DLY and SC-DLY groups (Fig. 2C). The Chao1, Pielou-e, and Shannon indices did not show significant differences between the CON-DLY and SC-DLY groups (*P* > 0.05, Fig. 2D). At the phylum level, the top 10 microbial communities were mainly composed of *Proteobacteria*, *Firmicutes*, *Bacteroidota*, *Euryarchaeota*, *Actinobacteriota*, *Planctomycetota*, *Fusobacteriota*, *Verrucomicrobiota*, *Desulfobacterota*, and *Chloroflexi* in both groups. Moreover, a significant increase in the abundance of *Proteobacteria* and a decrease in *Bacteroidota* were observed in the SC-DLY group compared to the CON-DLY group. At the genus level, the top 10 dominant genera included *Escherichia-Shigella*, *Acinetobacter*, *Streptococcus*, *Clostridium_sensu_stricto_1*, *Turicibacter*, *Terrisporobacter*, *Lysinibacillus*, *Solibacillus*, *Macrococcus*, and *Romboutsia*. Moreover, the abundance of *Acinetobacter* in the SC-DLY group was higher than that in the CON-DLY group, while the abundance of *Escherichia-Shigella* and *Streptococcus* was lower (Fig. 2E).

**Fig 3:**
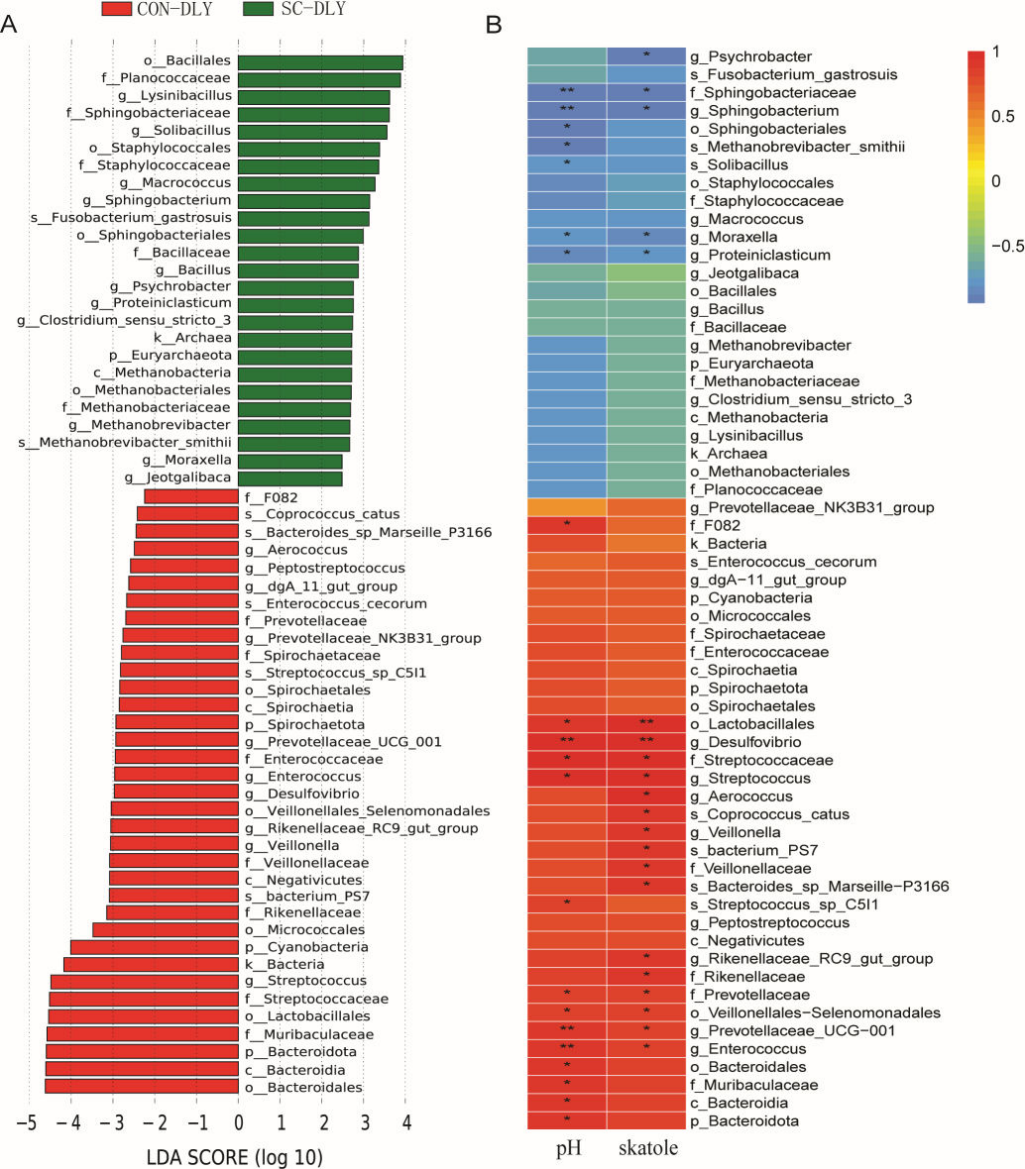
LEfSe and correlation analysis between CON-DLY and SC-DLY groups. (**A**) The histogram according to the linear discriminant analysis (LDA) value of LEfSe analysis. (**B**) Pearson correlation analysis between microbiotal communities and the value of pH and skatole. Red indicates a positive correlation, blue indicates a negative correlation, and * indicates statistically significant correlation (*P* < 0.05), ** indicates statistically highly significant correlation (*P* < 0.01).

As shown in Fig. 3A, linear discriminant analysis effect size (LEfSe) identified 35 differential microbial groups enriched in the CON-DLY group, including 1 kingdom, 3 phyla, 3 classes, 5 orders, 8 families, 10 genera, and 5 species. The SC-DLY group contained 25 enriched groups, including 1 kingdom, 1 phylum, 1 class, 4 orders, 5 families, 11 genera, and 2 species. At the genus level, the abundance of *Lysinibacillus*, *Sojibacillus*, *Macrococcus*, *Sphingobacterium*, *Fusobacterium_gastrosuis*, *Bacillus*, *Psychrobacter*, *Proteiniclasticum*, *Clostridium_sensu_stricto_3*, *Methanobrevibacter*, *Moraxella*, and *Jeotgalibaca* in the SC-DLY group was significantly higher than that in the CON-DLY group. Meanwhile, the expression abundance of *Aerococcus*, *Peptostreptococcus*, *dgA_11_gut_group*, *Prevotellaceae_NK3B31_group*, *Prevotellaceae_UCG_001*, *Enterococcus*, *Desulfovibrio*, *Rikenellaceae_RC9_gut_group*, *Veillonella*, and *Streptococcus* in the SC-DLY group was significantly lower than that in the CON-DLY group. The Spearman correlation between differential microbes and pH and skatole was analyzed. In total, 30 differential microbes were significantly correlated with pH and skatole at the genus level. *Sphingobacterium*, *Moraxella*, and *Proteiniclasticum* were significantly negatively correlated with pH and skatole, while *Desulfovibrio*, *Streptococcu*s, *Prevotellaceae_UCG_001*, and *Enterococcus* were significantly positively correlated with pH and skatole (Fig. 3B).

### *S. cerevisiae* changed tryptophan metabolism of *in vitro* fermentation solution

**Fig 4:**
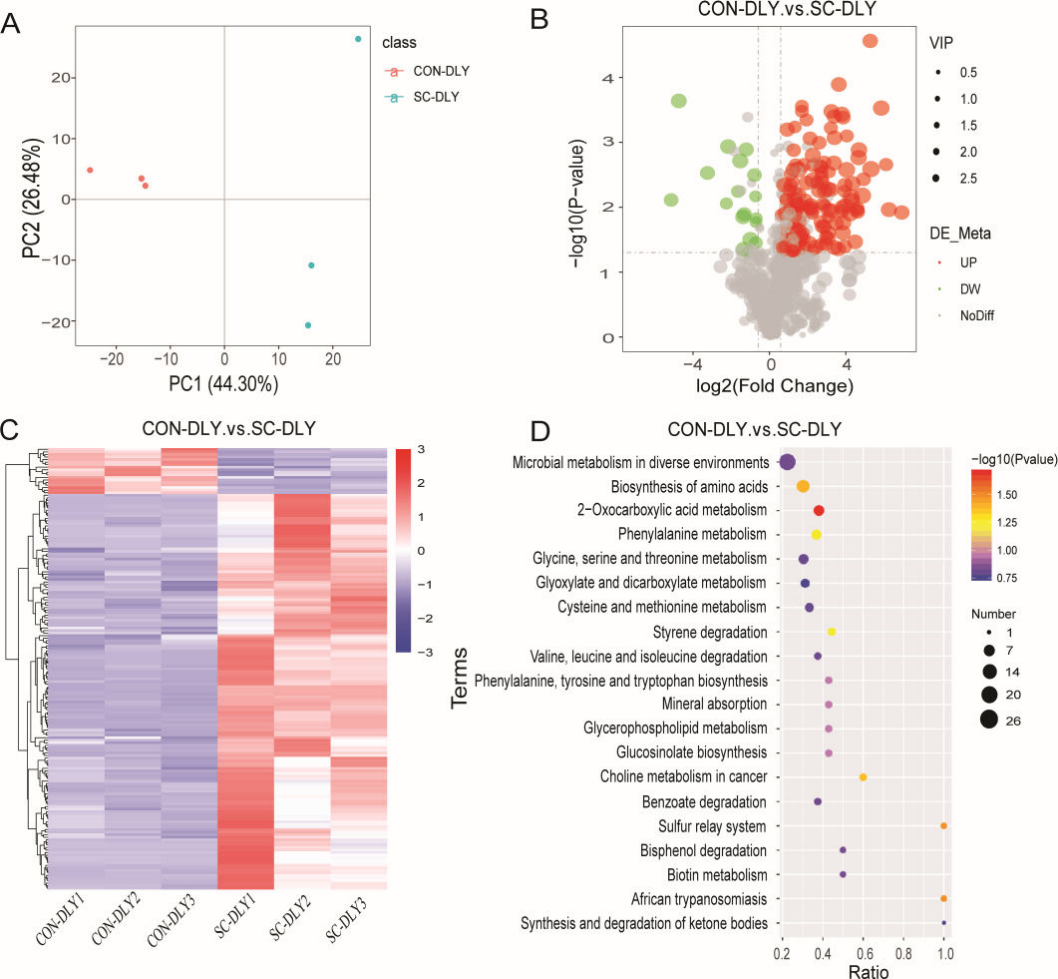
Analysis of differential metabolites of *in vitro* fermentation after *S. cerevisiae* supplementation. (**A**) Score plot of the PCA model between CON-DLY and SC-DLY groups. (**B**) Volcano plot of differential metabolites in the CON-DLY versus SC-DLY groups. Red, green, and gray indicate upregulated, downregulated, and non-significant metabolites, respectively. (**C**) Heatmap of 173 differential metabolites between CON-DLY and SC-DLY groups. An increase and a decrease in level are represented by red and blue, respectively. (**D**) KEGG enrichment of differential metabolites. The color of the point represented the *P* value of the hypergeometric test. The size of the point represented the number of differential metabolites in the pathway.

The principal components analysis (PCA) model showed that metabolites in the SC-DLY group were distinctly separated from those in the CON-DLY group (Fig. 4A). A total of 173 metabolites showed significant differences between the CON-DLY and SC-DLY groups. Among them, 154 metabolites were upregulated, and 19 metabolites were downregulated in SC-DLY relative to CON-DLY (Fig. 4B and C; Table S1).

These differential metabolites, based on KEGG enrichment analysis, were mainly involved in the top 10 metabolic pathways. These included microbial metabolism in diverse environments, biosynthesis of amino acids, 2-oxocarboxylic acid metabolism, phenylalanine metabolism, glycine, serine and threonine metabolism, glyoxylate and dicarboxylate metabolism, cysteine and methionine metabolism, styrene degradation, valine, leucine and isoleucine degradation, and phenylalanine, tyrosine, and tryptophan biosynthesis (Fig. 4D).

**Fig 5:**
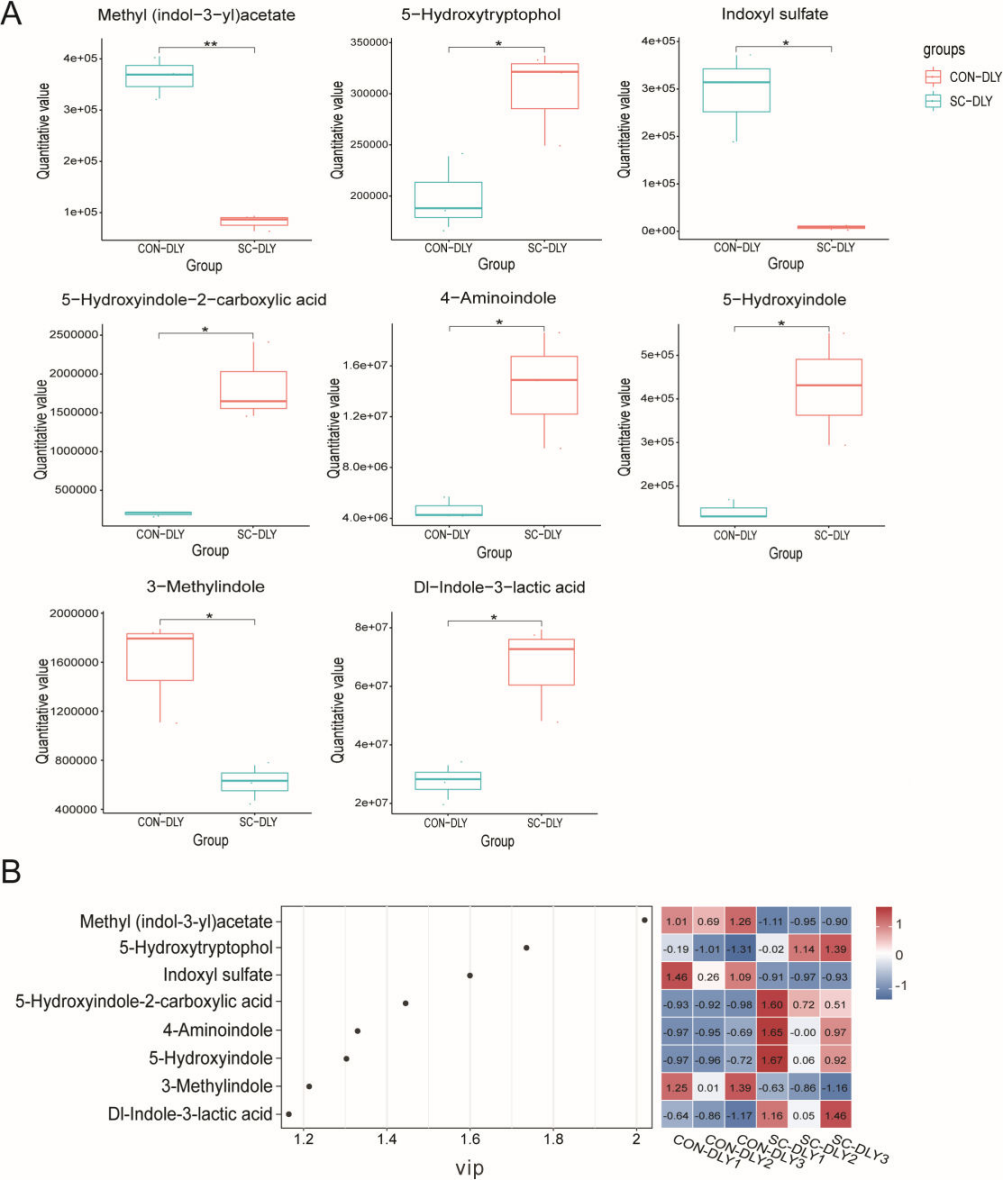
Eight differential indole derivatives in the CON-DLY and SC-DLY groups. (**A**) Box plots for indole derivatives levels. * Indicates a statistically significant difference between the SC-DLY and CON-DLY (*P* < 0.05), ** indicates a statistically highly significant difference between the SC-DLY and CON-DLY (*P* < 0.01). (**B**) VIP score analysis of indole derivatives. Red and blue indicate upregulated and downregulated metabolites, respectively.

**Fig 6:**
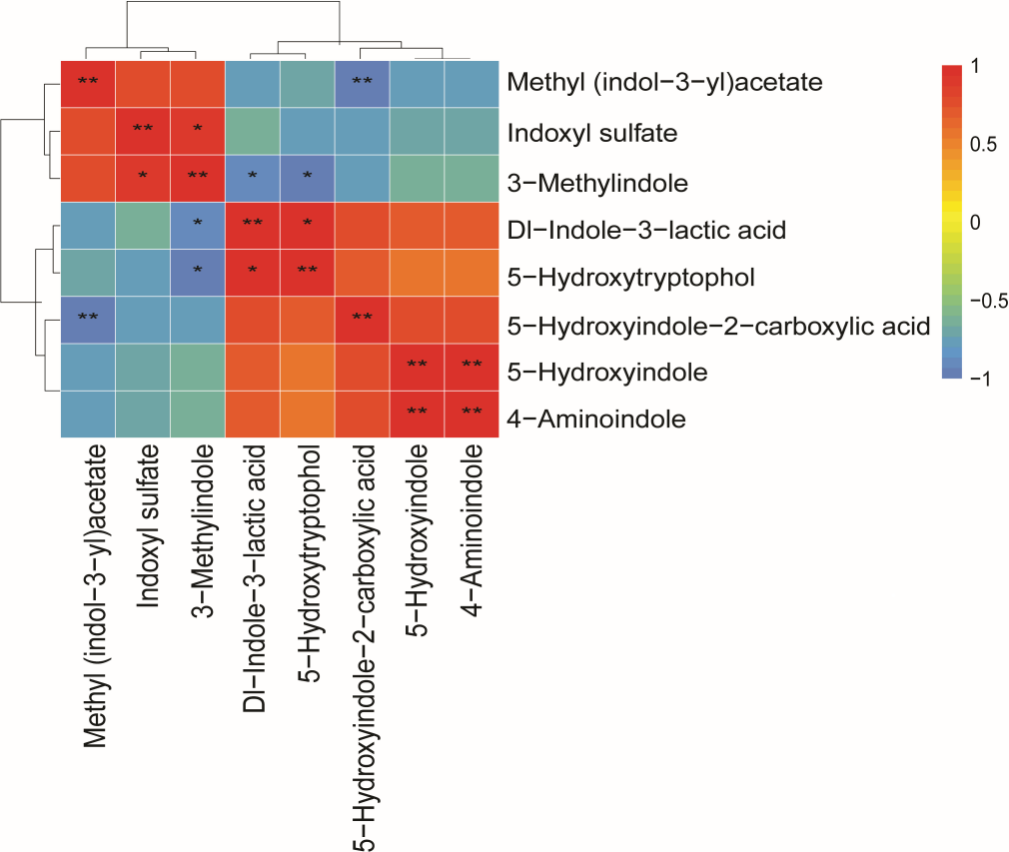
Pearson correlation analysis among differential indole derivatives. Red indicates a positive correlation, blue indicates a negative correlation, and * indicates statistically significant correlation (*P* < 0.05), ** indicates statistically highly significant correlation (*P* < 0.01).

A total of eight differential indole derivatives were identified between the CON-DLY and SC-DLY groups. The levels of methyl (indol-3-yl)acetate, indoxyl sulfate, and 3-methylindole were significantly decreased, while 5-hydroxyindole-2-carboxylic acid, Dl-indole-3-lactic acid, 5-hydroxyindole, 4-aminoindole, and 5-hydroxytryptophol were significantly increased after *S. cerevisiae* supplementation (Fig. 5A and B). These eight indole derivatives were subjected to correlation analysis. The results showed that the level of methyl (indol-3-yl)acetate was significantly negatively correlated with the level of 5-hydroxyindole-2-carboxylic acid (*P* < 0.01). The level of indoxyl sulfate was significantly positively correlated with the level of 3-methylindole (*P* < 0.05). The level of 3-methylindole was significantly negatively correlated with the levels of Dl-indole-3-lactic acid and 5-hydroxytryptophol (*P* < 0.05). The level of Dl-indole-3-lactic acid was significantly positively correlated with the level of 5-hydroxytryptophol (*P* < 0.01). The level of 5-hydroxyindole was significantly positively correlated with the level of 4-aminoindole (*P* < 0.05) (Fig. 6).

## DISCUSSION

*S. cerevisiae*, as a beneficial feed additive, enhances feed digestibility and growth performance, reduces pathogenic bacteria, and improves animal health (18). Furthermore, *S. cerevisiae* mitigates odor emissions from livestock and reduces the environmental impacts of livestock production. A previous study reported that *S. cerevisiae* had a strong effect on the removal of volatile gases in pig manure, with an elimination rate reaching 27.7% (23). Moreover, a *S. cerevisiae* composite inoculant significantly reduced ammonia, dimethyl sulfide, butyric acid, and isovaleric acid (24). In our study, during *in vitro* fermentation, the pH value decreased after *S. cerevisiae* addition and reached its lowest point at 24 h. In parallel, skatole concentration was also significantly reduced. Previous studies suggested that a decrease in pH leads bacteria to metabolize tryptophan into indole rather than skatole (25). Our results indicate that *S. cerevisiae* supplementation produced organic acids during fermentation, lowered pH, and reduced skatole levels in pig fermentation, which aligns with findings from other studies.

A previous study demonstrated that skatole production is regulated by multiple factors, including intestinal location, substrate availability, and gut microbiota. Among these, intestinal microbiota is recognized as the primary factor influencing skatole formation (26). Our research has revealed that *S. cerevisiae* altered the gut microbiome composition at the phylum level by increasing *Proteobacteria* and decreasing *Bacteroidota*. Previous research identified *Bacteroides* as a key contributor to skatole production in pig manure (27) and swine lagoon slurry (28). Additionally, a decrease in *Bacteroidota* has been associated with reduced skatole production (29).

The LDA analysis revealed that *Lysinibacillus*, *Sojibacillus*, *Macrococcus*, *Sphingobacterium*, *Fusobacterium_gastrosuis*, *Bacillus*, *Psychrobacter*, *Proteiniclasticum*, *Methanobrevibacter*, *Moraxella*, and *Jeotgalibaca* were significantly increased under *S. cerevisiae* treatment. In contrast, *Aerococcus*, *Peptostreptococcus*, *Prevotellaceae_NK3B31_group*, *Prevotellaceae_UCG_001*, *Enterococcus*, *Desulfovibrio*, *Veillonella*, and *Streptococcus* were significantly decreased at the genus level. According to Spearman’s correlation analysis, we determined an interaction pattern between gut microbes and host metabolites. The relative abundance of *Sphingobacterium*, *Moraxella*, and *Proteiniclasticum* was significantly negatively correlated with pH and skatole. In contrast, the relative abundance of *Desulfovibrio*, *Streptococcus*, *Prevotellaceae_UCG-001*, and *Enterococcu*s was significantly positively correlated with pH and skatole. Studies report that *Sphingobacterium* (30), *Lysinibacillus*, *Sojibacillus*, *Bacillus,* and *Psychrobacter* (31) act as beneficial bacteria in the gut, enhancing gut health and resistance to pathogens. Moreover, *Moraxella* exerts a protective effect and has been associated with decreased illness severity in infants hospitalized with bronchiolitis (32). An increase in *Proteiniclasticum* promotes short-chain fatty acid production (33), thereby lowering pH and reducing skatole concentration. Furthermore, studies show that *Desulfovibrio* and Streptococcus intensify the inflammatory response, worsening inflammatory bowel disease (34). The abundance of *Streptococcus* correlates with increased illness severity in bronchiolitis, reduces microbiome stability, and associates positively with high-risk bronchiolitis metabolites (32). The abundance of *Prevotellaceae* was significantly increased in diarrheal rats (35), and its high levels may disrupt mucosal barrier function and correlate positively with colonic inflammation (36). Prior research reported that magnolol addition binds to indole pyruvate decarboxylase of *Desulfovibrio*, reducing its abundance and decreasing skatole concentration. A significant positive correlation between *Desulfovibrio* and skatole has also been reported (26). Similarly, the β-glucan extracted from *S. cerevisiae* DPUL-C6, *Pichia kudriavzevii* DPUL-51-6Y, and *Kluyveromyces marxianus* DPUL-F15 increased *Lactobacillus*, *Bacteroides*, and *Turicibacter* populations and decreased *Erysipelotrichaceae-Clostridium*, *Ruminococcaceae-Ruminococcus*, *Desulfovibrio*, and *Streptococcus* (37). These changes are consistent with our observation that beneficial bacteria (*Bacillus*, *Sphingobacterium*, *Moraxella*, *Proteiniclasticum*, etc.) had high abundance, while harmful bacteria (*Desulfovibrio*, *Streptococcus*, etc.) and skatole concentrations were reduced after *S. cerevisiae* treatment. These microbiota shifts explain the mechanism by which *S. cerevisiae* contributes to skatole reduction.

The regulation of tryptophan metabolism is shaped by changes in intestinal microbiota. Based on our quasi-targeted metabolomics data, pathways involving microbial metabolism and amino acid metabolism were found to be enriched after *S. cerevisiae* supplementation. The downregulated levels of methyl (indol-3-yl)acetate, indoxyl sulfate, and 3-methylindole, and the upregulated levels of 5-hydroxyindole-2-carboxylic acid, DL-indole-3-lactic acid, 5-hydroxyindole, 4-aminoindole, and 5-hydroxytryptophol were observed in the SC-DLY group. The conversion of tryptophan to skatole mainly involves two steps. First, tryptophan is deaminated into indole-3-acetic acid. Second, indole-3-acetic acid is decarboxylated into skatole (38). Studies indicate that elevated levels of methyl (indol-3-yl)acetate, a derivative of indole-3-acetic acid (39), disrupt the tryptophan metabolic pathway and contribute to sleep-wake cycle disorders (40). Indoxyl sulfate acts as a harmful metabolite and uremic toxin that induces the necrosis of glomeruli and renal failure in uremic patients (41). Hydroxytryptophol, a serotonin metabolite, plays a key role as an intestinal neurotransmitter and is involved in gastrointestinal motility regulation (42). It is reported that 5-hydroxyindole-3-acetic acid has the ability to resist DSS-induced colitis by upregulating tight junction proteins, increasing the expression level of anti-inflammatory factors, and reducing the expression level of pro-inflammatory factors, thereby supporting intestinal integrity and alleviating inflammation (43). Indole-3-lactic acid helps maintain gut microbiota balance, improves intestinal inflammation, and inhibits colorectal cancer growth (44). A significant augment in indole-3-lactic acid and a negative correlation between skatole and indole-3-lactic acid were observed in pigs treated with mulberry leaves (14). Oral administration of β-glucan from *P. kudriavzevii* DPUL-51-6Y, *K. marxianus* DPUL-F15, and *S. cerevisiae* DPUL-C6 was shown to increase indole-3-β-acrylic acid, indole-3-lactic acid, and tryptophol levels, while reducing 3-indole glyoxylic acid and indole-3-acetamide in DSS-treated mice. This indicates that β-glucan from these three yeast strains reverses DSS damage and enhances indole derivative production (37). Based on Spearman’s correlation analysis, skatole (3-methylindole) shows a significant positive correlation with indoxyl sulfate and a negative correlation with DL-indole-3-lactic acid and 5-hydroxytryptophol, supporting previous findings. We speculate that *S. cerevisiae* supplementation promotes tryptophan conversion into beneficial catabolites, such as indole-3-lactic acid and 5-hydroxytryptamine, while reducing its conversion into harmful metabolites, such as methyl (indol-3-yl)acetate, indoxyl sulfate, and 3-methylindole in pigs. This observation further validates the impact of *S. cerevisiae* supplementation in reducing odorous compounds.

### Conclusion

This study demonstrates that *S. cerevisiae* supplementation significantly reduces skatole concentration during fermentation. Furthermore, skatole reduction is associated with the abundance of specific genera, such as *Sphingobacterium*, *Moraxella*, *Proteiniclasticum*, *Desulfovibrio*, and *Streptococcus*, and correlates with specific indole derivatives, such as indoxyl sulfate, DL-indole-3-lactic acid, and 5-hydroxytryptophol. These findings provide a theoretical reference for developing more effective microbial agents to reduce odor emissions.

## MATERIALS AND METHODS

### Animals and administration

Ten Duroc × Landace × Yorkshire pigs (five male and five female) with similar body weight (approximately 100 ± 5 kg) and good health were used as donors. The pigs were fed three times a day at 8:00, 12:00, and 20:00 and free to drink water. The feeding period was 30 days. The nutritive composition of the basal diet (post-growth and finishing pig compound feed from Zhengda Feed Co., Ltd.) is shown in Table 1.

**TABLE 1:** The nutritive composition of the basal diet

Items	H_2_O	Crude protein	Crude fiber	Coarse ash	Calcium		Total phosphorus	Sodium chloride		Lysine
Contents	≤14.0%	≥15.0%	≤7.0%	≤8.0%	≥0.60%	≤1.20%	≥0.50%	≥0.30%	≤0.80%	≥0.80%

### Activation and growth of *S. cerevisiae*

*S. cerevisiae* (ATCC 26497) was obtained from Beijing Beina Chuanglian Biotechnology Research Institute. After activation, 1 mL of the *S. cerevisiae* solution was diluted to 1 × 10⁸ CFU/mL, then inoculated into 100 mL of YPD (yeast extract 5 g, peptone 5 g, glucose 20 g, and maltose 2 g) and incubated at 38°C, 120 rpm. From 0 to 24 h, 1 mL of the bacterial solution was collected every 2 h, the absorbance value at 560 nm (OD₅₆₀) was measured by using a visible spectrophotometer (UV-5100H, Shanghai) under aseptic conditions, and a growth curve was drawn.

### Sample collection and preparation

Pig rectal contents were collected with a rectal swab under sterile conditions and were immediately transferred to sterilized airtight vials within 30 min. All rectal contents were blended, mixed with sterilized phosphate buffer (pH = 7.4; 1:4, wt/vol), and then filtered through four layers of sterile coarse cotton cloth to obtain rectal content homogenate as inoculum.

### Experimental design

Two groups were established as follows: CON-DLY (control group), 20 mL inoculum + 1 mL L-tryptophan (final concentration 250 μmol/L) + 5 mL sterile distilled water, and SC-DLY (treatment group), 20 mL inoculum + 1 mL L-tryptophan (final concentration 250 μmol/L) + 5 mL *S. cerevisiae* (1 × 10⁸ CFU/mL). Each group has three replicates. All fermentation solutions were incubated in a 250 mL fermentation flask at 38°C for 72 h with a slight agitation of 50 rpm, and CO_2_ was introduced throughout the whole process to keep it anaerobic.

### pH measurement

From 0 to 72 h, 1 mL of the fermentation solution was collected by using a sterile tip every 12 h. The pH value was measured immediately using a portable pH meter (Mettler Toledo, Stockholm, Sweden), which was calibrated using a standard solution before use.

### Detection of skatole

Fermentation solution samples from diverse environmental conditions were blended. A sample of 0.5 g was accurately weighed and mixed with 20 mL of methanol. After ultrasonic extraction for 30 min, the solution was filtered using the filter membrane with 0.45 μm. For chromatographic analysis, 5 μL of the sample was injected for detection. The chromatographic conditions were as follows: column, Syncronis C18 (5 μm, 4.6 × 250 mm; Thermo Fisher Scientific, USA); column temperature, 30°C; mobile phase, methanol/water = 55:45 (vol/vol); flow rate, 1.0 mL/min; excitation wavelength, 254 nm; emission wavelength, 350 nm; and sample size: 20 μL. Skatole analytical standards with 99% purity were purchased from Sigma-Aldrich Trading Co. Ltd. (Shanghai, China). The quantification of skatole content was performed using a high-performance liquid chromatograph from Thermo Fisher Scientific, USA.

### 16S rRNA sequencing

Total DNA was extracted from fermentation solution samples by using a DNA extraction kit (TIANamp Soil DNA Kit, Beijing TIANGEN Co., Beijing, China). The amount and integrity of the DNA products were estimated by using an agarose gel with 1.2% electrophoresis. Universal bacterial primers 515F (5′-GTGCCAGCMGCCGCGGTAA-3′) and 806R (5′-GGACTACHVGGGTWTCTAAT-3′) were used to amplify the V4 regions of the bacterial 16S rRNA. The PCR products were gel-purified, quantified, pooled, and sequenced using the Illumina NovaSeq platform (Biotechnology Co., Ltd., Beijing, China). After quality control filtration, all sequencing data were uploaded to the QIIME2 (Version QIIME2-202202) platform for analysis. The obtained sequences were clustered into OTUs by UPARSE 7.1, and the similarity was set at 97%. The alpha diversity (Chao1, Pielou-e, and Shannon indices) and PCoA of beta diversity by QIIME2 software were calculated. Community composition differences between groups were analyzed at different taxonomic levels. The significance test was conducted using LEfSe (LDA > 2, *P* < 0.05) to evaluate the composition and structure differences of microbia. This data is available at the National Center for Biotechnology Information database, where it has been assigned ID PRJNA1362983.

Spearman correlation analysis was performed between the abundance of differential microbiota and the content of skatole.

### Metabolomics analysis

The liquid chromatography–tandem mass spectrometry (LC-MS/MS) platform provided by Novogene Co., Ltd. (Beijing, China) was used for quasi-targeted metabolomics analysis of fermentation solution samples. Firstly, 100 mg of the sample was ground in liquid nitrogen, and the homogenate was resuspended in methanol. Next, the samples were placed on ice for 5 min and centrifuged for 20 min at 15,000 × *g* at 4°C. Subsequently, the supernatant was collected for dilution and subjected to further centrifugation. The final solution was used for analysis in the LC-MS/MS system and paired with an ExionLC AD system (SCIEX) coupled with a QTRAP 6500+ mass spectrometer (SCIEX). The parameters for the positive ion model of LC-MS/MS system were set as follows: curtain gas: 35 psi; collision gas: medium; IonSpray voltage: 5,500 V; temperature: 500°C; ion source gas 1: 55; and ion source gas 2: 55. The parameters for the negative ion model were set as follows: curtain gas: 35 psi; collision gas: medium; IonSpray voltage: −4,500 V; temperature: 500°C; ion source gas 1: 55; and ion source gas 2: 55. Raw data files resulting from this analysis were processed using Compound Discoverer 3.1 (CD3.1, Thermo Fisher). Annotation of metabolites was performed using the KEGG, HMDB, and LIPID MAPS databases. PCA was conducted using MetaX software to obtain the VIP value of each metabolite. The differential metabolites were analyzed using single-variable analysis (*t*-test), with the specific parameters, VIP >1, *P* value <0.05, and fold change >1.2 (or <0.833). The volcano map was generated using the R package ggplot2, which combined the VIP, log₂ (fold change), and log₁₀ (*P* value) values to screen metabolites. The cluster heatmap data were normalized using z-scores of differential metabolite intensity values, with visualization conducted using the pheatmap package in R. The KEGG database was used to investigate the functions and metabolic pathways associated with these metabolites. This data is available at the MetaboLights database, where it has been assigned ID REQ20260102215763. Spearman correlation analysis was performed to determine the significant correlations among the differential metabolites.

### Statistical analysis

Skatole concentrations and pH values were analyzed by Student’s *t*-test using SPSS 20.0 (IBM Corp., Armonk, NY, USA). Data are presented as mean ± standard deviation (SD) for each group. Statistical significance was defined as *P* <0.05. In Spearman correlation analysis, *P* <0.05 and the correlation coefficient |r| >0.5 were considered significant. Adobe Illustrator CC (https://www.adobe.com/) was used to manipulate figures and arrange groups.

## Data Availability

The 16S rRNA sequence data is available at the National Center for Biotechnology Information (NCBI) database, under accession no. PRJNA1362983.

## SUPPLEMENTAL MATERIAL

Table S1 spectrum.03292-25-s0001.pdf Qualitative and quantitative list of different metabolites.

ASM does not own the copyrights to Supplemental Material that may be linked to, or accessed through, an article. The authors have granted ASM a non-exclusive, world-wide license to publish the Supplemental Material files. Please contact the corresponding author directly for reuse.
