# Supplementary material for: Effects of Saccharomyces cerevisiae on the regulation of skatole, microorganisms, and tryptophan metabolites in vitro fermentation of pig
Source: Microbiol Spectr. 2026 Feb 27;14(4):e03292-25. doi: 10.1128/spectrum.03292-25 (PMC13055380; doi:10.1128/spectrum.03292-25)
Supplement: Table S1 — Qualitative and quantitative list of different metabolites. [file spectrum.03292-25-s0001.pdf]

Table S1 Qualitative and quantitative list of different metabolites

Name	Formula	FC	log2FC	Pvalue	VIP
Methyl nicotinate	$C_7H_7NO_2$	26.859	4.747	0.000	1.996
Methyl (indol-3-yl)acetate	$C_{11}H_{11}NO_2$	4.488	2.166	0.001	2.020
N alpha-Acetyl-L-Arginine	$C_8H_{16}N_4O_3$	2.320	1.214	0.001	1.762
cis-Aconitic acid	$C_6H_6O_6$	2.885	1.528	0.002	2.101
Shikimic Acid	$C_7H_{10}O_5$	9.487	3.246	0.003	1.709
3-Amino-4-methylpentanoic acid	$C_6H_{13}NO_2$	1.723	0.785	0.003	1.627
Diethylglutarate	$C_9H_{16}O_4$	0.065	-3.935	0.006	1.382
13,14-dihydro-15-keto-PGD2	$C_{20}H_{32}O_5$	3.136	1.649	0.006	1.356
Pyridoxamine	$C_8H_{12}N_2O_2$	1.657	0.728	0.007	1.082
Indoxyl sulfate	$C_8H_7NO_4S$	35.467	5.148	0.008	1.601
Homo-Gamma-Linolenic Acid (C20:[illegible]	$C_{20}H_{34}O_2$	4.771	2.254	0.009	1.164
Benzenebutanoic acid	$C_{10}H_{12}O_2$	2.495	1.319	0.013	1.761
Deoxycorticosterone acetate	$C_{23}H_{32}O_4$	2.608	1.383	0.014	1.937
2-Hydroxyphenylacetic acid	$C_8H_8O_3$	1.610	0.687	0.015	1.067
4-Hydroxy-3-methylbenzoic acid	$C_8H_8O_3$	1.637	0.711	0.017	1.091
acetoacetate	$C_4H_6O_3$	1.997	0.998	0.031	2.087
Urea	CH_4N_2O	1.642	0.715	0.035	1.408
6-Hydroxydopamine	$C_8H_{11}NO_3$	2.554	1.353	0.044	2.021
L-Kynurenine	$C_{10}H_{12}N_2O_3$	2.131	1.092	0.050	1.250
Phenylacetylglycine	$C_{10}H_{11}NO_3$	0.026	-5.277	0.000	1.875
Hippuric acid	$C_9H_9NO_3$	0.081	-3.635	0.000	2.008
3-(3-Hydroxyphenyl)propanoic acid	$C_9H_{10}O_3$	0.308	-1.697	0.000	1.372
Lysopc 14:0	$C_{22}H_{46}NO_7P$	0.017	-5.854	0.000	2.264
N,N-Dimethylglycine	$C_4H_9NO_2$	0.106	-3.239	0.000	1.948
Methyl 3-Hydroxyphenylacetate	$C_9H_{10}O_3$	0.308	-1.698	0.000	1.366
Adrenochrome	$C_9H_9NO_3$	0.073	-3.784	0.000	2.307
Choline	$C_5H_{14}NO+$	0.095	-3.393	0.000	1.870
N-acetyl-glutamate	$C_7H_{11}NO_5$	0.069	-3.864	0.000	1.694
2-Aminophenol	C_6H_7NO	0.261	-1.940	0.000	1.427
Prolylhydroxyproline	$C_{10}H_{16}N_2O_4$	0.398	-1.331	0.001	1.302
5-Hydroxyindole-2-carboxylic acid	$C_9H_7NO_3$	0.108	-3.217	0.001	1.446
Xanthurenic Acid	$C_{10}H_7NO_4$	0.526	-0.928	0.001	1.836
L-Hydroxyproline	$C_5H_9NO_3$	0.060	-4.054	0.001	1.410
Choline Hydroxide	$C_5H_{15}NO_2$	0.094	-3.404	0.001	1.957
4-Methyl-2-pentanone	$C_6H_{12}O$	0.229	-2.128	0.001	1.709
N-acetyl-L-ornithine	$C_7H_{14}N_2O_3$	0.164	-2.607	0.001	1.645
1-Palmitoyl-Sn-Glycero-3-Phosphoch	$C_{24}H_{50}NO_7P$	0.039	-4.687	0.001	2.216

D-Glutamine	$C_5H_{10}N_2O_3$	0.415	-1.268	0.001	1.593
Hydantoin-5-propionic acid	$C_6H_8N_2O_4$	0.207	-2.272	0.002	2.111
Lysopc 16:0	$C_{24}H_{50}NO_7P$	0.039	-4.667	0.002	2.202
N-Acetylalanine	$C_5H_9NO_3$	0.136	-2.874	0.002	1.462
5-oxoproline	$C_5H_7NO_3$	0.097	-3.372	0.002	2.126
3-Hydroxypropanoic acid	$C_3H_6O_3$	0.015	-6.107	0.002	1.489
Dihydrouracil	$C_4H_6N_2O_2$	0.285	-1.811	0.002	1.658
2-Phenylacetamide	C_8H_9NO	0.386	-1.375	0.002	1.522
N,N'-diacetylchitobiose	$C_{16}H_{28}N_2O_{11}$	0.199	-2.328	0.002	1.758
N-Formyl-L-aspartate	$C_5H_7NO_5$	0.154	-2.696	0.002	1.316
alpha-Benzylsuccinic acid	$C_{11}H_{12}O_4$	0.159	-2.657	0.002	2.059
Lysopc 16:1	$C_{24}H_{48}NO_7P$	0.025	-5.319	0.003	2.587
Val-Ser	$C_8H_{16}N_2O_4$	0.347	-1.529	0.003	2.453
3,4-Dihydroxybutyrate	$C_4H_8O_4$	0.425	-1.234	0.003	1.758
Pyridoxine 5'-Phosphate	$C_8H_{12}NO_6P$	0.061	-4.044	0.003	2.391
1-Methylnicotinamide	$C_7H_9N_2O+$	0.150	-2.735	0.003	1.378
Carnitine-C3	$C_{10}H_{17}NO_4$	0.115	-3.123	0.003	1.259
alpha-D-Glucose	$C_6H_{12}O_6$	0.465	-1.105	0.003	1.319
L-Serine	$C_3H_7NO_3$	0.152	-2.722	0.004	1.653
N-Propionylglycine	$C_5H_9NO_3$	0.033	-4.925	0.004	1.656
2-Hydroxy-3-methylbutanoic acid	$C_5H_{10}O_3$	0.207	-2.273	0.004	2.253
L-Lysine	$C_6H_{14}N_2O_2$	0.436	-1.197	0.004	1.580
4-Hydroxy-2-oxoglutaric acid	$C_5H_6O_6$	0.179	-2.482	0.004	1.367
5-Aminopentanoate	$C_5H_{11}NO_2$	0.149	-2.746	0.004	1.922
2-Piperidinone	C_5H_9NO	0.260	-1.942	0.004	2.023
Imatinib	$C_{29}H_{31}N_7O$	0.049	-4.343	0.004	2.097
Prostaglandin B2	$C_{20}H_{30}O_4$	0.064	-3.955	0.005	1.804
Homotaurine	$C_3H_9NO_3S$	0.585	-0.774	0.005	1.999
Cyclohexylsulfamate	$C_6H_{13}NO_3S$	0.462	-1.113	0.005	1.500
2-Hydroxybutyric acid	$C_4H_8O_3$	0.120	-3.060	0.005	2.222
Trimethylamine	C_3H_9N	0.107	-3.230	0.005	1.895
L-Fucose	$C_6H_{12}O_5$	0.487	-1.038	0.005	1.410
Hydroxypyruvic acid	$C_3H_4O_4$	0.165	-2.604	0.006	2.142
L-Pyroglutamic acid	$C_5H_7NO_3$	0.135	-2.889	0.006	1.907
L-Citrulline	$C_6H_{13}N_3O_3$	0.398	-1.328	0.006	1.875
1-Amino-propan-2-ol	C_3H_9NO	0.341	-1.551	0.006	1.565
Anserine	$C_{10}H_{16}N_4O_3$	0.034	-4.866	0.007	2.541
Lysopc 17:0	$C_{25}H_{52}NO_7P$	0.052	-4.269	0.007	2.562

DL-Citrulline	$C_6H_{13}N_3O_3$	0.415	-1.268	0.007	1.393
3,4-Dihydroxymandelaldehyde	$C_8H_8O_4$	0.549	-0.865	0.007	1.204
Propionyl-L-carnitine	$C_{10}H_{19}NO_4$	0.377	-1.407	0.008	1.123
Barbituric acid	$C_4H_4N_2O_3$	0.270	-1.886	0.008	1.465
Cytarabine	$C_9H_{13}N_3O_5$	0.534	-0.906	0.008	1.627
4-Hydroxyhippuric acid	$C_9H_9NO_4$	0.050	-4.324	0.008	2.081
L-Histidinol	$C_6H_{11}N_3O$	0.365	-1.455	0.009	1.465
N-Acetylputrescine	$C_6H_{14}N_2O$	0.145	-2.791	0.009	1.269
Alloxan	$C_4H_4N_2O_5$	0.135	-2.892	0.009	1.356
S-Methyl-5'-thioadenosine	$C_{11}H_{15}N_5O_3S$	0.223	-2.164	0.009	2.005
Ala-gly	$C_5H_{10}N_2O_3$	0.377	-1.409	0.009	1.540
O-Succinyhomoserine	$C_8H_{13}NO_6$	0.283	-1.819	0.009	1.164
D-Glucosamine 6-phosphate	$C_6H_{14}NO_8P$	0.367	-1.447	0.009	1.690
Lysopc 18:1	$C_{26}H_{52}NO_7P$	0.065	-3.951	0.009	2.079
11-Ketoetiocholanolone	$C_{19}H_{28}O_3$	0.327	-1.610	0.010	1.722
1-Oleoyl-Sn-Glycero-3-Phosphocholi	$C_{26}H_{52}NO_7P$	0.072	-3.803	0.010	2.024
Hydroquinone	$C_6H_6O_2$	0.221	-2.177	0.010	2.016
cAMP	$C_{10}H_{12}N_5O_6P$	0.343	-1.545	0.010	1.029
4-Hydroxyphenylacetate	$C_8H_8O_3$	0.482	-1.054	0.010	1.074
LysoPC 18:0	$C_{26}H_{54}NO_7P$	0.041	-4.596	0.010	1.662
L-Alanine	$C_3H_7NO_2$	0.229	-2.126	0.011	1.112
Thiamine	$C_{12}H_{17}N_4OS+$	0.013	-6.257	0.011	2.075
L-Erythrulose	$C_4H_8O_4$	0.118	-3.082	0.011	1.930
Urocanic acid	$C_6H_6N_2O_2$	0.435	-1.200	0.011	1.156
1-Stearoyl-Sn-Glycerol-3-Phosphoch	$C_{26}H_{54}NO_7P$	0.042	-4.584	0.011	1.677
5'-Deoxy-5'-(methylthio)adenosine	$C_{11}H_{15}N_5O_3S$	0.212	-2.240	0.011	2.061
Lysopc 18:2	$C_{26}H_{50}NO_7P$	0.107	-3.231	0.011	2.061
L-Tryptophan	$C_{11}H_{12}N_2O_2$	0.396	-1.336	0.011	1.191
2-Deoxy-D-galactose	$C_6H_{12}O_5$	0.071	-3.811	0.011	1.767
PAF C-16	$C_{26}H_{54}NO_7P$	0.041	-4.616	0.012	1.664
UDP	$C_9H_{14}N_2O_{12}P_2$	0.008	-6.929	0.012	1.899
SDMA	$C_8H_{18}N_4O_2$	0.558	-0.842	0.012	1.961
Sarcosine	$C_3H_7NO_2$	0.213	-2.233	0.013	1.148
Dl-Indole-3-lactic acid	$C_{11}H_{11}NO_3$	0.412	-1.278	0.013	1.166
Lysopc 18:3	$C_{26}H_{48}NO_7P$	0.053	-4.228	0.013	2.108
NG,NG-Dimethyl-L-arginine	$C_8H_{18}N_4O_2$	0.640	-0.644	0.013	1.446
3-Methylindole	C_9H_9N	2.561	1.357	0.014	1.214
Glycylglycine	$C_4H_8N_2O_3$	0.402	-1.314	0.014	1.736

Butylmalonic acid	$C_7H_{12}O_4$	0.117	-3.097	0.014	1.292
5-Hydroxyindole	C_8H_7NO	0.336	-1.572	0.015	1.304
4-Aminoindole	$C_8H_8N_2$	0.328	-1.607	0.016	1.331
gamma-Muurolene	$C_{15}H_{24}$	0.397	-1.333	0.017	1.401
Gentisic acid	$C_7H_6O_4$	0.155	-2.692	0.017	1.616
5-Hydroxylysine	$C_6H_{14}N_2O_3$	0.069	-3.850	0.017	2.271
Lysopc 20:4	$C_{28}H_{50}NO_7P$	0.090	-3.477	0.018	1.977
Adipamide	$C_6H_{12}N_2O_2$	0.147	-2.763	0.020	1.686
N-Methylalanine	$C_4H_9NO_2$	0.138	-2.861	0.020	1.652
Porphobilinogen	$C_{10}H_{14}N_2O_4$	0.365	-1.453	0.021	1.061
Dithranol	$C_{14}H_{10}O_3$	0.450	-1.151	0.022	1.579
Methionine	$C_5H_{11}NO_2S$	0.327	-1.611	0.022	1.182
L-Leucyl-L-Alanine	$C_9H_{18}N_2O_3$	0.599	-0.739	0.023	1.211
Bz-RS-ISer(3-Ph)-Ome	$C_{17}H_{17}NO_4$	0.505	-0.985	0.023	1.814
Allantoin	$C_4H_6N_4O_3$	0.433	-1.207	0.024	1.297
Octopamine	$C_8H_{11}NO_2$	0.827	-0.275	0.024	1.377
L-Rhamnose monohydrate	$C_6H_{14}O_6$	0.364	-1.459	0.024	1.774
Oxaloacetate	$C_4H_4O_5$	0.119	-3.073	0.024	1.953
dADP	$C_{10}H_{15}N_5O_9P_2$	0.070	-3.837	0.025	2.115
3-Methylphenylacetic acid	$C_9H_{10}O_2$	0.294	-1.766	0.025	1.300
2-Phenylpropionic acid	$C_9H_{10}O_2$	0.310	-1.691	0.028	1.202
(S)-2-Aminobutanoate	$C_4H_9NO_2$	0.127	-2.978	0.029	1.595
Nelarabine	$C_{11}H_{15}N_5O_5$	0.478	-1.066	0.030	1.013
Tetrahydrobiopterin	$C_9H_{15}N_5O_3$	0.295	-1.763	0.030	1.621
Glutaric Acid	$C_5H_8O_4$	0.119	-3.068	0.030	1.871
4-Hydroxy-3-methoxyphenylglycol	$C_9H_{12}O_4$	0.558	-0.841	0.030	1.031
Ureidosuccinic acid	$C_5H_8N_2O_5$	0.325	-1.621	0.031	1.651
4-Ethoxy-4-oxobutanoic acid	$C_6H_{10}O_4$	0.412	-1.281	0.032	2.120
Dimethyl fumarate	$C_6H_8O_4$	0.045	-4.485	0.034	1.982
Sulfoacetic acid	$C_2H_4O_5S$	0.577	-0.793	0.035	1.479
all-trans-Retinal	$C_{20}H_{28}O$	0.238	-2.072	0.035	1.810
D-2-Aminobutyric acid	$C_4H_9NO_2$	0.130	-2.939	0.037	1.550
Guanidinoacetic acid	$C_3H_7N_3O_2$	0.154	-2.695	0.037	1.230
5-Hydroxytryptophol	$C_{10}H_{11}NO_2$	0.657	-0.605	0.038	1.737
D-Phenylalanine	$C_9H_{11}NO_2$	0.428	-1.223	0.038	1.042
Cyclocytidine	$C_9H_{11}N_3O_4$	0.447	-1.161	0.039	1.005
isoleucine	$C_6H_{13}NO_2$	0.423	-1.243	0.039	1.135
Isoquinoline	C_9H_7N	0.347	-1.527	0.039	1.745

L-Leucine	$C_6H_{13}NO_2$	0.411	-1.281	0.039	1.069
L-allo-Isoleucine	$C_6H_{13}NO_2$	0.429	-1.221	0.040	1.074
S-adenosyl-L-methioninamine	$C_{15}H_{22}N_6O_5S$	0.066	-3.930	0.040	1.696
4-Hydroxyphenylpyruvate	$C_9H_8O_4$	0.560	-0.835	0.041	1.386
Dimethylmalonic acid	$C_5H_8O_4$	0.187	-2.422	0.041	1.983
Decanoic acid	$C_{10}H_{20}O_2$	0.299	-1.741	0.041	2.073
N-Formylglycine	$C_3H_5NO_3$	0.621	-0.686	0.041	1.248
cis-7-Hexadecenoic Acid	$C_{16}H_{30}O_2$	0.095	-3.403	0.042	2.045
Pantetheine	$C_{11}H_{22}N_2O_4S$	0.397	-1.333	0.043	1.466
2'-O-methylinosine	$C_{11}H_{14}N_4O_5$	0.082	-3.609	0.044	1.861
Glycyl-L-leucine	$C_8H_{16}N_2O_3$	0.421	-1.247	0.045	1.034
6-Methoxy-2-naphthoic acid	$C_{12}H_{10}O_3$	0.567	-0.819	0.045	1.212
Gly-Phe	$C_{11}H_{14}N_2O_3$	0.390	-1.357	0.045	1.402
Lactic acid	$C_3H_6O_3$	0.592	-0.755	0.045	1.699
Stearic acid	$C_{18}H_{36}O_2$	0.202	-2.310	0.046	1.125
2,5-Dihydroxypyridine	$C_5H_5NO_2$	0.524	-0.933	0.047	1.374
3-Methyl-2-oxovaleric acid	$C_6H_{10}O_3$	0.405	-1.305	0.047	1.149
cis-4-Hydroxy-D-proline	$C_5H_9NO_3$	0.348	-1.523	0.047	1.094
3-Hydroxyphenylacetic acid	$C_8H_8O_3$	0.441	-1.181	0.047	1.392
N-Acetylproline	$C_7H_{11}NO_3$	0.827	-0.273	0.047	1.035
2-Pyrrolidinone	C_4H_7NO	0.356	-1.490	0.048	1.143
Glycochenodeoxycholic acid	$C_{26}H_{43}NO_{5.}Na$	0.421	-1.247	0.049	1.013
